# Editorial: The impact of liquid biopsies in the management of liver cancer

**DOI:** 10.3389/fonc.2022.1083355

**Published:** 2022-11-29

**Authors:** Ismail Labgaa

**Affiliations:** Department of Visceral Surgery, Lausanne University Hospital (CHUV), University of Lausanne (UNIL), Lausanne, Switzerland

**Keywords:** hepatocellular carcinoma, cholangiocarcinoma, bile, plasma, CtDNA, CTC (circulation tumor cells), exosomes

Epidemiological data are unequivocal: liver cancer incidence and cancer-related mortality are raising and estimations predict >1 million of deaths by 2030, reflecting a major health problem (1). Primary liver cancer mainly encompasses hepatocellular carcinoma (HCC) –its most common form- and cholangiocarcinoma (CCA). Intensive efforts have been pursued to better control the burden of these diseases and to improve their treatments. As an example, this led to the approval of a number of new systemic therapies in advanced HCC after a decade tainted by a single recommended agent and numerous negative randomized clinical trials (RCT). The Barcelona Clinic Liver Cancer (BCLC) strategy acts as the backbone of HCC management, and was recently updated (2). This algorithm, like other alternative ones, essentially relies on clinical variables aiming to reflect tumor burden, allowing to stratify patients and to *in fine* recommend specific treatments for each stage. Although undeniably valuable for clinicians, these classifications fail to encapsulate the biological complexity of HCC –that combines two diseases in most cases, with cirrhosis and cancer- and may thus be misleading in the decision-making for treatment allocation (3). As an illustration, patients with intermediate stage (BCLC-B) must undergo loco-regional treatments (LRT) like trans-arterial chemo-embolization (TACE) while data showed that a subset of these patients may rather benefit from surgery, a treatment option that is not yet recommended in the armamentarium of BCLC-B HCC (4, 5).

Liquid biopsy, the molecular analysis of by-products released into the bloodstream by solid tumors (i.e. DNA, RNA, cells and exosomes), has emerged as a major breakthrough in research and management of cancers. In comparison with other cancers, data on liquid biopsy in liver malignancies remain scant, particularly for CCA. However, the available studies provided very encouraging results, highlighting multiple applications including surveillance (6), diagnosis (7), treatment selection (8) and even prognostication (9).

This Research Topic entitled “*The Impact of Liquid Biopsies in the Management of Liver Cancer*” includes original articles and a review, and provides novel data and new insights on the recent progresses observed in the field. Circulating tumor cells (CTC) and their prognostic value have been evidenced in many cancers, including in HCC. First, studies provided the proof-of-concept to detect CTC, enumerate them and analyze their association with prognosis (10). Later, technologies like single-cell sequencing allowed deciphering the complexity of these analytes (11). Three original studies of the present issue permitted to better characterize HCC-CTCs and therewith to better understand their clinical significance and potential applications. Chen et al. analyzed a specific subgroup of CTC associated with white blood cells cluster and referred as CTC-WBC, in a cohort of 136 HCC patients. CTC-WBC were detected in 32% of patients and correlated with other prognostic factors such as microvascular invasion, portal vein thrombus or extrahepatic metastasis. Moreover, it showed a good ability to discriminate metastatic disease, with an AUC of 0.821 and was associated with poorer outcomes: higher recurrence rate and shorter recurrence-free survival (RFS). Yan et al. investigated the role of FGL1 –the main ligand of the immune checkpoint LAG-3- which appeared as an interesting candidate. Precisely, they performed immunofluorescence staining of isolated CTC in 109 HCC patients. FGL1+ CTC were detected in 37% of cases and were associated with higher rate of distant metastasis, more advanced TNM stage and lower survival, compared to patients with FGL1- CTC. In addition, 8 out of 10 patients with FGL1+ CTC treated with PD-1/PD-L1 blockade agents showed treatment resistance. Su et al. explored PD-L1 expression of CTC in a cohort of 47 HCC patients receiving PD-1 inhibitor, radiotherapy and antiangiogenic agent. The analyzes suggested that PD-L1+ CTC count may be used as a predictor of response for this triple therapy. Finally, Gonvers et al. performed a comprehensive review of the literature for the available data on liquid biopsy in patients undergoing liver transplant (LT) for HCC. The study revealed rare but promising results, underpinning the importance and the need to further investigate how liquid biopsy may contribute to improve the management of LT for HCC.

Two original articles explored liquid biopsies in CCA. The first one provided a proof-of-principle to study circulating free DNA (cfDNA) in bile. CfDNA has been essentially studied in plasma, including for liver cancers (12, 13). Data on bile-derived liquid biopsies are very rare (14). In their study, Li et al. reported the feasibility of next-generation sequencing (NGS) in bile cfDNA, showing that it reliably recapitulated the genomic aberrations identified in tissue samples from the tumors. Fründt et al. explored whether CTC detected preoperatively may be associated with worse outcomes. As anticipated, they showed impaired survival in this subgroup of patients, indicating that it may be a biomarker of existing metastases that may help distinguishing patients who may benefit from surgical resection from those at high risk of early recurrence.

Despite a meteoric rise, several challenges and unmet needs still exist in the field of liquid biopsy. One of them is the necessity to intensify basic research and to generate new models, in order to better understand the underlying mechanisms of circulating analytes releases (15, 16). Another is to conduct integrative analysis of circulating analytes (e.g. CTC and cfDNA), in order to optimize liquid biopsies performance.

Articles included in this Research Topic confirmed the promises and hope raised by the noteworthy performance of liquid biopsies in liver cancer. Researchers in the field must push the envelope towards veritable precision medicine. The current practice in cancers management including liver malignancies aims to answer questions such as: “which treatment should be recommended in a particular subgroup of patients?”, defining subgroups based on tumor burden, like mentioned above for the BCLC strategy. However, one may think the other way around and ask: “which patient will benefit from this particular treatment?”, regardless of tumor burden but based on genomic alterations. With this perspective, liquid biopsies appear as ideal tools to shift this paradigm.

## Author contributions

Study concept and design: IL Acquisition of data: IL Analysis and interpretation of data: not applicable Drafting of the manuscript: IL Critical revision of the manuscript for important intellectual content: IL.

## Conflict of interest

The author declares that the research was conducted in the absence of any commercial or financial relationships that could be construed as a potential conflict of interest.

## Publisher’s note

All claims expressed in this article are solely those of the authors and do not necessarily represent those of their affiliated organizations, or those of the publisher, the editors and the reviewers. Any product that may be evaluated in this article, or claim that may be made by its manufacturer, is not guaranteed or endorsed by the publisher.
